# Management of diabetic macular edema: from anti-VEGF to emerging therapies

**DOI:** 10.3389/fmed.2026.1761325

**Published:** 2026-03-24

**Authors:** Meng Ji, Wei Wei, Hui Gong, Guisen Zhang

**Affiliations:** 1 Affiliated Inner Mongolia Clinical College of Inner Mongolia Medical University, Hohhot, Inner Mongolia Autonomous Region, China; 2 Ophthalmology Department, Inner Mongolia Autonomous Region People’s Hospital, Hohhot, Inner Mongolia Autonomous Region, China; 3 Inner Mongolia Chaoju Eye Hospital, Inner Mongolia Chaoju Institute of Eye Disease Control, Hohhot, Inner Mongolia Autonomous Region, China

**Keywords:** anti-vascular endothelial growth factor, diabetic macular edema, diabetic retinopathy, photocoagulation, systemic treatment

## Abstract

Diabetic macular edema (DME) is a frequent and vision-threatening complication of diabetes, arising from a complex interplay of inflammatory, vascular, and metabolic pathways. The advent of anti–vascular endothelial growth factor (anti-VEGF) therapy has fundamentally transformed DME management and is now established as the cornerstone of treatment. In this review, we provide an updated overview of current and emerging therapeutic approaches for DME. We summarize the clinical evidence supporting established anti-VEGF agents, including ranibizumab, aflibercept, and conbercept, as well as the complementary roles of intravitreal corticosteroids and subthreshold micropulse laser therapy. Particular emphasis is placed on recent advances, such as dual-pathway inhibition with faricimab, the potential retinal benefits of systemic agents including sodium–glucose cotransporter-2 inhibitors and metformin, and novel strategies aimed at improving treatment durability, including long-acting anti-VEGF formulations and gene-based therapies. Together, these developments reflect a shift toward more sustained, individualized, and mechanism-based management of DME.

## 1 Introduction

The global prevalence of diabetes mellitus (DM) has been rising steadily, with approximately 1 in 10 adults aged 20–79 years currently living with the disease (1). Diabetes has become an increasingly serious public health concern worldwide (2), and its complications severely affect patients’ quality of life, especially in visual health. It is estimated that approximately 93 million people worldwide are affected by diabetic retinopathy (DR) (3, 4). Diabetic macular edema (DME) is one of the common complications in diabetic patients. In recent years, intravitreal injection of anti-vascular endothelial growth factor (VEGF) drugs has been globally recognized as the most effective treatment for disease (5, 6). DME is a multifactorial disorder driven by hypoxia, chronic inflammation, increased vascular permeability, and dysregulated angiogenesis. Although DME may develop at any stage of DR, it most commonly occurs during the early to moderate phases of the disease. Persistent hyperglycemia, a hallmark of diabetes, induces upregulation of VEGF, leading to breakdown of the blood–retinal barrier, enhanced vascular leakage, and pathological neovascular responses. These alterations impair retinal perfusion, exacerbate tissue hypoxia, and perpetuate inflammatory signaling. The resulting cascade of inflammatory mediators further damages retinal vascular and neural structures, increases retinal vascular permeability, and ultimately culminates in macular edema.

## 2 Treatment of diabetic macular edema

### 2.1 Anti-VEGF therapy

A substantial body of clinical evidence has identified VEGF as a key pathogenic mediator in both DME and proliferative diabetic retinopathy (PDR). Inhibition of VEGF effectively suppresses pathological neovascularization and reduces retinal vascular permeability, thereby alleviating macular edema and improving visual outcomes. Accordingly, anti-VEGF therapy is widely recommended as the first-line treatment for DME, a position endorsed by the 2022 Chinese Clinical Guidelines for Diabetic Retinopathy. Currently available anti-VEGF agents for DME management include Ranibizumab (Lucentis®, Genentech, United States/Novartis, Switzerland), Aflibercept (Eylea®, Regeneron, United States/Bayer, Germany), Conbercept (Lumitin®, Chengdu Kanghong Biotech, China), Bevacizumab (Avastin®, Genentech, United States), Faricimab (Vabysmo®, Genentech, United States/Roche, Switzerland), and Brolucizumab (Beovu®, Novartis, Switzerland).

#### 2.1.1 Ranibizumab

Ranibizumab is a 48-kDa recombinant humanized monoclonal antibody Fab fragment that binds and neutralizes all biologically active isoforms of VEGF-A. It is produced using engineered *Escherichia coli* expression systems. Owing to its relatively small molecular size, ranibizumab demonstrates efficient retinal tissue penetration, a pharmacokinetic characteristic that supports its widespread use in the treatment of diabetic macular edema (7). A hypoxia-inducible glycoprotein, VEGF-A orchestrates endothelial cell migration, vascular hyperpermeability, and neovascularization (8, 9). Ranibizumab can block the interaction between VEGF-A and its receptors, reducing vascular permeability, angiogenesis, and endothelial cell proliferation (10).

**Figure 1:**
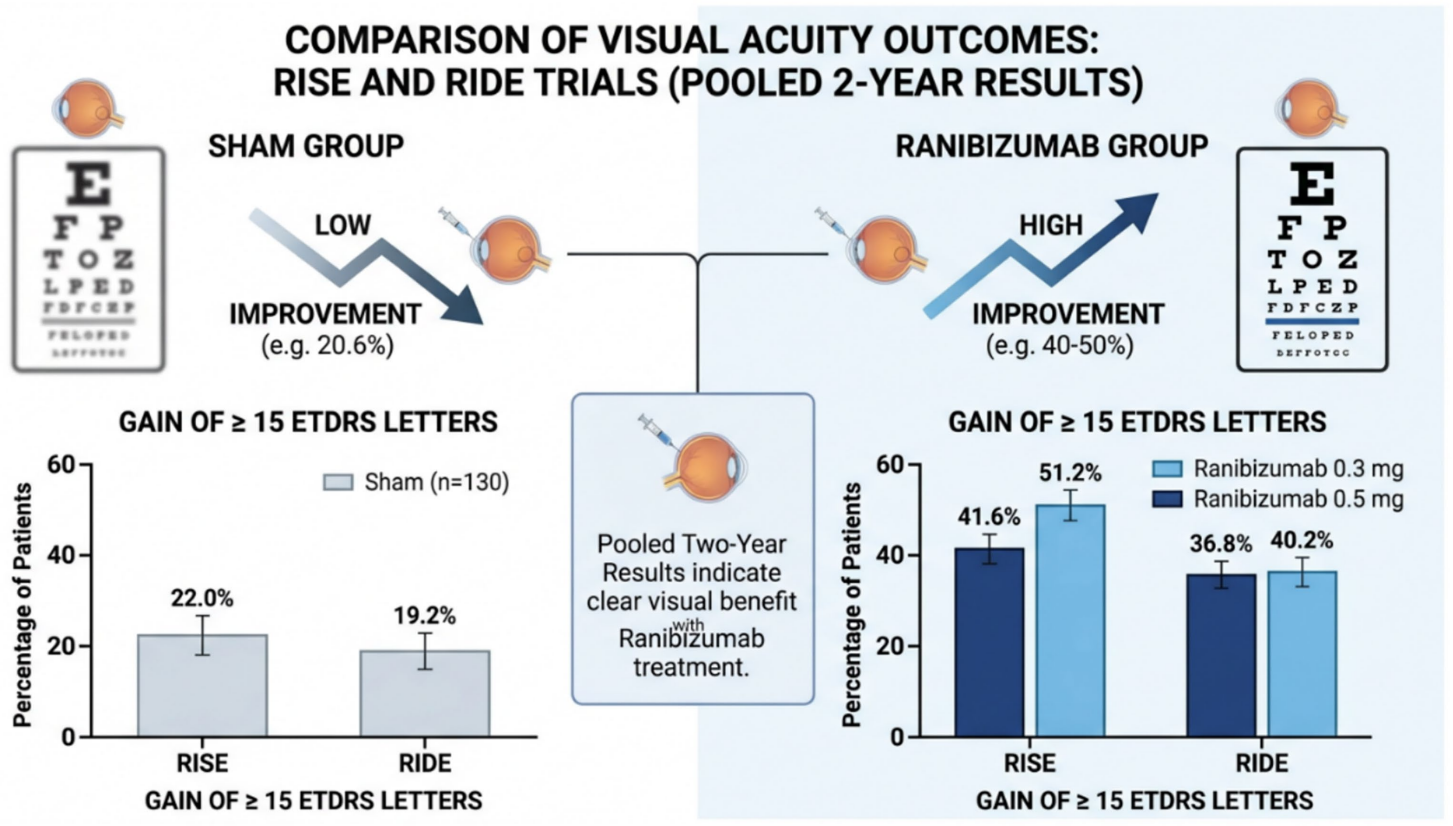
Schematic illustration showing Comparison of Visual Acuity Outcomes: RISE and RIDE Trials (Pooled 2-YEAR Results).

RISE and RIDE (11) are two similar randomized, double-blind, controlled Phase III clinical studies. Participants were randomized to receive intravitreal ranibizumab at doses of 0.3 mg or 0.5 mg, or sham injections, administered monthly over a two-year period. Pooled two-year results from the pivotal phase III RISE and RIDE trials demonstrated a clear visual benefit associated with ranibizumab treatment. In the RISE study, gains of at least 15 ETDRS letters were observed in 51.2% of eyes treated with 0.3 mg and 41.6% treated with 0.5 mg, compared with 22.0% in the sham group; similar patterns were reported in RIDE, with corresponding proportions of 36.8, 40.2, and 19.2%, respectively (Figure 1). In addition to superior visual outcomes, ranibizumab substantially reduced the need for rescue macular laser therapy in both trials. During the subsequent pro re nata phase, most patients required a median of four to five injections to maintain treatment benefits, regardless of baseline diabetic retinopathy severity, underscoring the importance of continued monitoring and sustained therapy to preserve disease stability.

#### 2.1.2 Aflibercept

Aflibercept is a 115 kDa recombinant fusion protein composed of two protein fragments (VEGFR-1 and VEGFR-2) of the VEGF receptor and the crystallizable fragment of human immunoglobulin G1 (12). It has a strong affinity for VEGF-A, VEGF-B, and placental growth factor (PIGF). PIGF is another member of the VEGF family and has the potential to induce retinal neovascularization (13). Therefore, aflibercept not only inhibits the pathological effects of VEGF but also suppresses the angiogenic effects of PIGF.

A 24-month head-to-head randomized trial demonstrated that aflibercept, bevacizumab, and ranibizumab all produced significant visual acuity improvements in eyes with diabetic macular edema; however, treatment efficacy varied according to baseline visual acuity. Among eyes with poorer pre-treatment vision (approximately 20/50 or worse), aflibercept was associated with greater visual gains compared with the other agents (14).

Two parallel phase III randomized, double-blind trials, VIVID and VISTA, provided robust evidence supporting the efficacy and safety of aflibercept in patients with clinically significant diabetic macular edema (15). Both studies employed an initial loading phase followed by intravitreal aflibercept 2 mg administered either every 4 weeks or every 8 weeks. Extended follow-up demonstrated that visual benefits were largely maintained through 100 weeks with both dosing schedules. Notably, the every-8-week regimen produced visual outcomes comparable to monthly treatment, suggesting that longer dosing intervals may effectively reduce injection burden without compromising efficacy.

Another study on aflibercept 8 mg, PULSAR, is a phase 3, randomized, three-arm, double-masked, non-inferiority, 96-week trial (16). It showed that, compared with aflibercept 2 mg, aflibercept 8 mg can improve treatment outcomes and provide sustained disease control in patients with neovascular age-related macular degeneration (nAMD).

#### 2.1.3 Conbercept

Conbercept, a next-generation anti-VEGF agent akin to aflibercept, binds every isoform of VEGF-A, VEGF-B and PIGF, and has won NMPA approval for DME, neovascular age-related macular degeneration and pathologic myopia-driven choroidal neovascularization (17, 18). Its affinity for VEGF is 50 times that of bevacizumab and 30 times that of ranibizumab (19, 20). Compared with aflibercept, it has a lower VEGF dissociation rate and higher binding affinity, resulting in a longer clearance time (21).

The 12-month multicenter, randomized, double-blind parallel controlled SAILING trial (22) aimed to evaluate the safety and efficacy of conbercept for the treatment of DME patients. The primary endpoint of this study was the change in best-corrected visual acuity (BCVA) from baseline to the 12-month follow-up, assessed using standardized ETDRS charts. Secondary endpoints included the safety profile of the treatment (evaluated by the incidence and severity of adverse events), changes in central macular thickness (CMT) measured by optical coherence tomography (OCT), and monitoring of retinal microstructural alterations during the treatment period. The results demonstrated that both BCVA and CMT were significantly improved in the conbercept group. At month 12, the mean BCVA in the conbercept group increased by 8.2 ± 9.5 letters from baseline, showing a statistically significant improvement (*p* < 0.001), whereas no significant change was observed in the laser group (*p* = 0.810). By month 12, 25% of patients in the conbercept group achieved a BCVA gain of ≥15 letters, compared with 14.9% in the laser group. Previous studies have highlighted the importance of injection frequency during the loading phase, generally indicating that a higher number of injections results in better BCVA and CMT outcomes.

A multicenter study comparing 3 + PRN and 6 + PRN regimens found that the 6 + PRN group exhibited more favorable efficacy trends and demonstrated significant improvements in the deep capillary plexus (DCP) over 1 year (23). Age, baseline HbA1c level, CMT, and FAZ area were significantly correlated with the outcomes of IVC injections.

#### 2.1.4 Bevacizumab

Bevacizumab is effective and cost-effective, and is used by many DME patients worldwide. Bevacizumab is a humanized monoclonal IgG antibody featuring dual antigen-binding domains that specifically target and neutralize VEGF-A (24). The T-Protocol randomized clinical trial compared the clinical efficacy of bevacizumab, ranibizumab, and aflibercept for the treatment of DME (14). During the 24-month follow-up, no outcome differences emerged among the three groups for eyes with better baseline BCVA. However, in eyes with poorer baseline vision, the aflibercept group demonstrated superior BCVA gains versus bevacizumab. While bevacizumab may exhibit marginally reduced efficacy compared to aflibercept for DME, its cost-effectiveness remains a viable treatment alternative.

#### 2.1.5 Faricimab

Faricimab is a bispecific monoclonal antibody targeting VEGF-A and angiopoietin-2 (Ang-2), with a molecular weight of 149 kDa. The drug can simultaneously inhibit VEGF-A and Ang-2 (25). The angiopoietin–Tie signaling pathway, consisting of the growth factors Ang-1 and Ang-2, which contain immunoglobulin-like and epidermal growth factor–like domains, plays a central role in maintaining retinal vascular stability, regulating angiogenesis, and controlling vascular permeability. Under normal physiological conditions, Ang-1 activates Tie2 receptor signaling, promoting endothelial cell survival and reinforcing intercellular junctions to preserve vascular integrity. In the context of retinal vascular disease, however, elevated Ang-2 functions as a competitive antagonist by displacing Ang-1 from Tie2, thereby impairing the protective Ang-1/Tie2 signaling axis and contributing to vascular dysfunction (26). Ang-1 activates Tie2 receptor signaling to enhance vascular integrity, while Ang-2 antagonizes this pathway by competitively binding Tie2, resulting in disrupted endothelial junctions (27).

**Figure 2:**
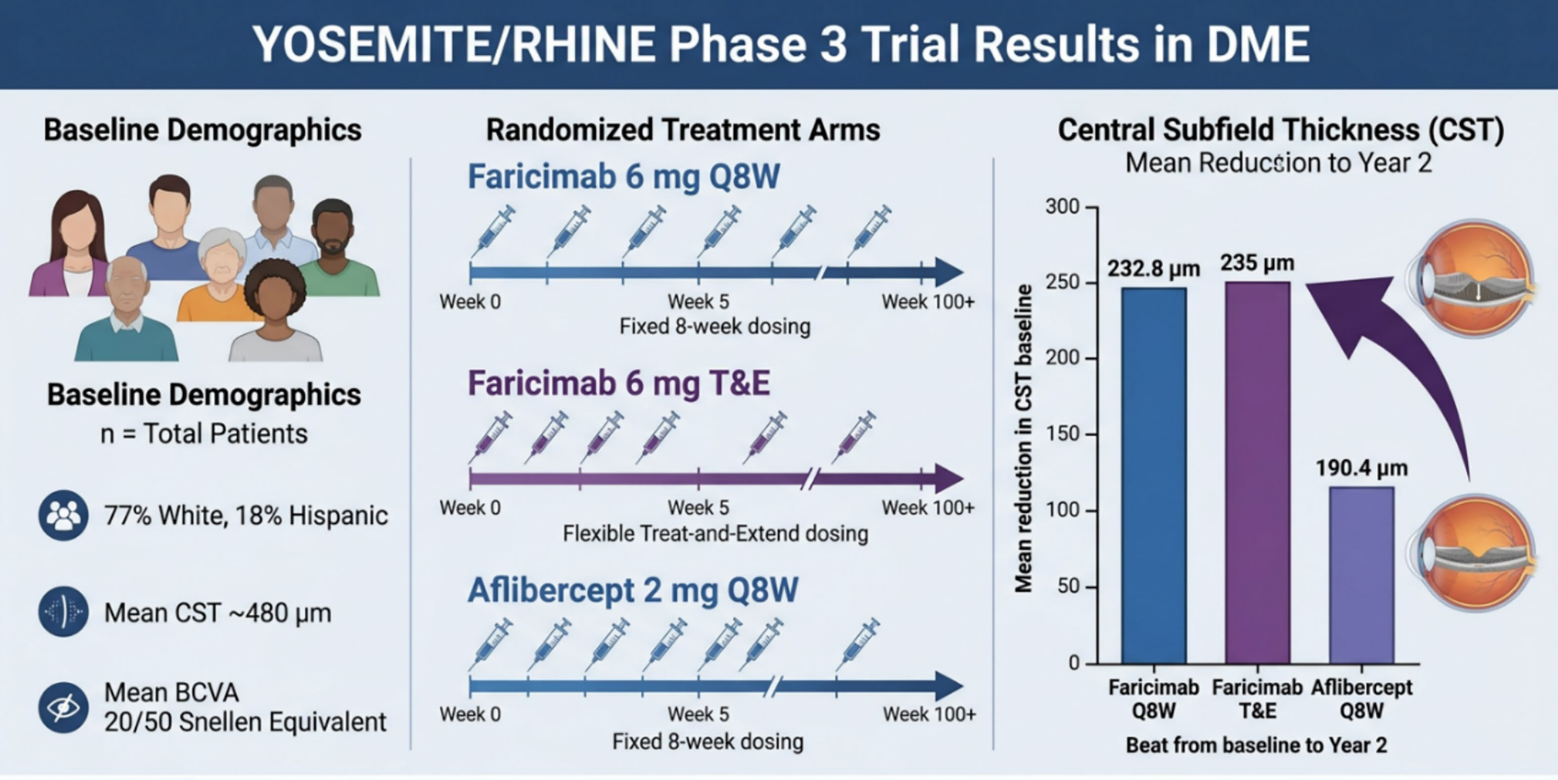
Schematic illustration showing YOSEMITE/RHINE Phase 3 Trial Results in DME.

Year-1 data from the phase 3 YOSEMITE and RHINE studies showed that mean best-corrected visual-acuity gains were similar across treatment arms in both trials, indicating that faricimab and aflibercept were roughly equivalent in improving patients’ vision. The more critical endpoint, however, was change in central subfield thickness (CST). In YOSEMITE, the faricimab Q8W arm achieved a 232.8 μm reduction in CST at year 1, significantly greater than the 190.4 μm reduction seen with aflibercept (*p* < 0.0001). In the faricimab treat-and-extend arm, CST decreased by 217.4 μm (*p* = 0.0004), again significantly better than aflibercept. In RHINE, faricimab Q8W and treat-and-extend arms also outperformed afliberpect, with CST reductions of 214.2 μm and 206.6 μm, respectively (*p* = 0.0006 and *p* = 0.0116), confirming faricimab’s superiority in reducing retinal thickness (28)(Figure 2).

#### 2.1.6 Brolucizumab

Brolucizumab is a humanized monoclonal antibody that selectively targets VEGF-A, incorporating a single-chain Fc fragment to inhibit VEGF-A binding to both VEGFR-1 and VEGFR-2. With a molecular weight of approximately 26 kDa—substantially lower than that of ranibizumab—it demonstrates improved tissue penetration. This distinctive pharmacological profile, combining low molecular weight with a relatively high therapeutic dose (6.0 mg), enhances bioavailability and prolongs the duration of therapeutic effect compared with conventional anti-VEGF agents (29). The KESTREL and KITE studies compared the efficacy and safety of brolucizumab and aflibercept for DME (30), mainly observing the changes in BCVA from pre-treatment to week 52. The KITE study showed that brolucizumab required fewer injections than aflibercept and its clinical efficacy was not inferior to aflibercept. Yet no head-to-head trial has directly compared faricimab with brolucizumab, leaving their relative merit unresolved.

### 2.2 Local corticosteroid therapy

Local corticosteroid therapy is an effective option for managing DME and can lead to improvements in BCVA. However, its use is commonly associated with adverse effects, most notably accelerated cataract formation and increased intraocular pressure. Notably, in pseudophakic eyes, corticosteroid treatment has been shown to be as effective as anti-VEGF therapy (31). Corticosteroid agents commonly used for DME include dexamethasone (DEX), fluocinolone acetonide (FA), and triamcinolone acetonide (TA). DEX is administered as a sustained-release intravitreal implant, FA is available as a long-acting implant, and TA can be delivered via intravitreal or subtenon injection. Comparative studies evaluating DEX implants, FA implants, and TA injections have demonstrated that all three corticosteroid formulations effectively manage DME. Furthermore, higher doses were associated with greater improvements in BCVA (32). Long-term, repeated, or high-dose use of corticosteroids can lead to cataract formation and elevated intraocular pressure, and may even result in permanent vision loss. Therefore, careful monitoring for complications is essential during their use.

#### 2.2.1 Intravitreal injection of triamcinolone acetonide

Intravitreal injection of triamcinolone acetonide (IVTA) can be used in cases where traditional macular laser therapy is ineffective. A randomized, double-masked, prospective study demonstrated that IVTA provides effective and acceptably safe short-term management for DME (33). High doses of TA are beneficial for improving CMT and BCVA. Clinical studies have shown that IVTA doses of ≥8 mg can significantly prolong the duration of visual acuity improvement in patients with DME. However, this benefit must be weighed against the increased risk of corticosteroid-related adverse effects, particularly cataract progression and elevated intraocular pressure (IOP), necessitating careful monitoring (34).

#### 2.2.2 Subtenon injection of triamcinolone acetonide

Although IVTA is effective in treating DME, intravitreal injections can be associated with complications such as acute infectious endophthalmitis and pseudo-phakic endophthalmitis. Subtenon triamcinolone acetonide (STTA) has been employed to manage Irvine–Gass syndrome and uveitis-related macular edema, with early studies suggesting its efficacy in DME as well. IVTA has been shown to produce significant improvements in BCVA and central retinal thickness (CRT) during the first 3 months. However, by 6 months, therapeutic equivalence was observed between IVTA and alternative treatments, indicating a decline in treatment durability and necessitating retreatment for all participants (35).

#### 2.2.3 Intravitreal sustained-release steroid implants

Both dexamethasone and fluocinolone acetonide function as effective intravitreal sustained-release steroid implants, with extensive clinical evidence supporting their therapeutic efficacy in DME. Research indicates that optimized dosing regimens demonstrate superior treatment outcomes compared to lower-dose alternatives, though with careful monitoring required for corticosteroid-related complications such as elevated IOP and cataract progression (36, 37). Following administration of the 0.5 μg/day fluocinolone acetonide (FA) implant, mean BCVA gains relative to baseline were 7.5, 6.9, and 5.7 letters at 3, 6, and 12 months, respectively. By contrast, patients receiving the 0.2 μg/day FA implant experienced mean BCVA improvements of 5.1, 2.7, and 1.3 letters at the same time points, demonstrating lower clinical efficacy compared with the higher-dose implant.

### 2.3 Laser photocoagulation

#### 2.3.1 Focal/grid laser

Focal/grid laser photocoagulation, established as the gold standard for DME following its 1985 efficacy validation, is clinically termed conventional laser therapy (38). However, adverse reactions such as choroidal neovascularization (39), enlargement of laser scars (40), and subretinal fibrosis (41) can occur after focal/grid laser photocoagulation. Studies have shown that grid photocoagulation can effectively control diabetic macular edema and has been widely used. However, retinal photocoagulation can damage retinal photoreceptors, increase the risk of decreased central vision and reduced dark adaptation, which are complications that should be noted. Figueira et al. (42) found that laser scars produced after conventional laser treatment were more numerous than those after subthreshold micropulse laser treatment. Vujosevic et al. (43) also mentioned that central retinal sensitivity improved in eyes treated with subthreshold micropulse laser but worsened in those treated with conventional laser. Clinical evidence from major trials confirms anti-VEGF therapy’s superiority over laser interventions for DME, leading to reduced utilization of focal/grid photocoagulation and its reclassification as a secondary treatment option (31).

#### 2.3.2 Subthreshold micropulse laser

Subthreshold micropulse laser photocoagulation (SMLP) can provide pulses lasting microseconds (44, 45), limiting the diffusion of heat to adjacent retinal and choroidal layers, thereby reducing thermal damage and scarring. SMLP is a laser emission technique in which the standard continuous-wave (CW) laser emission is interrupted by a series of repeated micropulses, with relatively long intervals between each micropulse to reduce tissue temperature (46, 47). The typical exposure time is 200 ms, with a duty cycle of 5%, and the energy level should be adjusted according to retinal thickness and titrated energy. Repeated micropulses can create temperature peaks and significant temperature differences in the tissue, while the pulse intervals allow the tissue temperature to cool between adjacent pulses, thereby minimizing heat accumulation (48). Compared with conventional laser photocoagulation, SMLP may offer superior efficacy and safety in improving BCVA, reducing CMT, and preserving contrast sensitivity. Clinically, SMLP is regarded as a safe and effective treatment for DME.

### 2.4 Vitrectomy

Prior to the advent of anti-VEGF therapy, pars plana vitrectomy served as a primary surgical intervention for diabetic macular edema (DME) management, demonstrating both clinical efficacy and broad adoption. Lewis et al. (49) reported on the efficacy of vitrectomy for the treatment of DME. Studies have reported that among 10 eyes exhibiting macular thickening and traction from the posterior vitreous membrane, 9 achieved improved visual outcomes following vitrectomy. This has led to the view that vitrectomy can alleviate macular traction, a key factor contributing to DME improvement. However, with the well-established efficacy of anti-VEGF therapy, vitrectomy is now typically reserved for selected cases, as current consensus designates anti-VEGF agents as the first-line treatment. Emerging evidence indicates that anti-VEGF therapy may provide superior outcomes even in eyes with vitreomacular traction or a thickened posterior hyaloid membrane (50).

Therefore, considering the risk of complications, initial anti-VEGF treatment remains the preferred option. Surgery is needed for those who find it difficult to control edema after injection or who have macular pucker and traction.

### 2.5 Systemic therapies

#### 2.5.1 Sodium-glucose cotransporter-2 inhibitors

Sodium-glucose cotransporter-2 inhibitors (SGLT2i) promote the excretion of glucose in urine by inhibiting the reabsorption of glucose by SGLT2. SGLT2i can lower blood glucose to treat diabetes and have also been proven to have a protective effect on the retina (51, 52). SGLT2 exhibits dual localization, being expressed not only in renal proximal tubules but also in bovine retinal pericytes, suggesting potential extrarenal physiological roles (53). A study by Matthews et al. (54) showed that SGLT2i can reduce retinal abnormalities, vascular leakage, and VEGF expression associated with diabetic retinopathy in model mice. SGLT2 inhibitors ameliorate DR and DME through glycemic control and osmotic diuresis, with potential direct retinal effects in diabetes.

#### 2.5.2 Metformin

Metformin is a major drug for treating diabetes, which can lower blood glucose levels without causing hypoglycemia (55). Metformin modulates VEGF-A mRNA splicing to preferentially generate the VEGF120 isoform, consequently attenuating VEGFR2 signaling activation through ligand-receptor interaction reduction (56). This agent suppresses VEGF-A protein synthesis through microRNA-mediated post-transcriptional regulation, wherein induced specific microRNAs selectively bind and destabilize VEGF-A mRNA transcripts (57). Metformin exerts neuroprotective effects on photoreceptors through AMP-activated protein kinase (AMPK) pathway activation, thereby preserving visual function in retinal degenerative conditions (58). Uwimana et al. (59) demonstrated that concurrent metformin and anti-VEGF therapy significantly lowers treatment resistance incidence in diabetic macular edema (DME) cases.

#### 2.5.3 Combined traditional Chinese and Western medicine treatment

Traditional Chinese ophthalmology has formed a unique method of syndrome differentiation and treatment based on long-term clinical practice experience. Integrating traditional Chinese medicine with Western therapeutic approaches demonstrates synergistic benefits in enhancing treatment outcomes for DME patients. Studies have shown that the combination of Xiaozhong Granules and Conbercept (60) and the combination of Wei’s Macular Edema Formula Granules and Ranibizumab (61) can effectively improve BCVA and CMT in DME patients, delay the recurrence of macular edema, improve systemic symptoms in patients, and have clinical safety. The combination of Modified Wuling Shenqi Decoction and macular grid photocoagulation can also improve retinal hemodynamics, reduce systemic inflammation, and regulate lipid metabolism disorders, thereby improving clinical symptoms and vision (62). However, due to the limitations of the study, the findings should be interpreted with caution and require validation by randomized controlled trials.

### 2.6 Emerging therapies

#### 2.6.1 OCS-01

OCS-01 is a topical DEX suspension developed by Oculis that employs innovative solubilizing nanoparticle technology to enhance the drug’s bioavailability and durability. This non-invasive formulation reduces the risks associated with intravitreal injections. In a multicenter, randomized, double-masked, vehicle-controlled phase II trial, 12-week data showed that the OCS-01 group achieved a significantly greater reduction in CMT and a higher proportion of patients gained ≥10 and ≥15 letters of vision. OCS-01 was generally well tolerated; however, ocular hypertension was noted in 21.2% of treated eyes versus none in the vehicle group. An ongoing phase III program is further evaluating OCS-01’s efficacy in DME (63).

#### 2.6.2 RGX-314

RGX-314 represents an innovative gene-based intervention for retinal neovascular disorders, utilizing AAV8 vectors to deliver genetic material encoding anti-VEGF antibody fragments directly to retinal cells through single-dose subretinal/suprachoroidal administration, enabling sustained intraocular anti-VEGF production. The ongoing ALTITUDE Phase II trial is evaluating the safety profile of suprachoroidally administered RGX-314 gene therapy for DR treatment (64).

#### 2.6.3 4D-150

4D Molecular Therapeutics recently announced positive topline results from the SPECTRA trial of 4D-150 in patients with DME (64). 4D-150 is a dual-mechanism gene therapy that employs the R100 vector to co-deliver DNA encoding aflibercept and a VEGF-C-targeting RNAi, enabling simultaneous blockade of the four key angiogenic factors—VEGF-A, -B, -C and PlGF—for the treatment of wet age-related macular degeneration (wet AMD) and DME.

Data showed that all 22 treated patients tolerated 4D-150 well; no intra-ocular inflammation was observed at any visit, underscoring a favorable safety profile. Efficacy was durable and dose-dependent, with sustained improvements in visual acuity and anatomical outcomes. Relative to the approved aflibercept 2 mg every-8-week regimen, the planned phase 3 dose cut treatment burden by 78%. Moreover, patients assigned to the phase 3 recommended-dose cohort required 58% fewer supplemental injections than those in the low-dose cohort, and four of the nine patients in the recommended-dose group achieved zero supplemental injections through week 60.

## 3 Conclusion

The management of DME has progressed markedly from conventional laser therapy to an era dominated by anti-VEGF agents. Nevertheless, the mechanisms of anti-VEGF non-response are diverse. In a subset of patients, a chronic inflammatory–fibrotic phenotype predominates, in which VEGF is no longer the principal driver. In addition, severe retinal ischemia and hypoxia can up-regulate bypass pathways such as PDGF and Ang-2, rendering anti-VEGF therapy ineffective. The high treatment burden and the presence of anti-VEGF–resistant patients have prompted the exploration of novel therapeutic strategies. Future DME management is likely to emphasize more individualized and durable approaches. Promising directions include targeting novel pathways such as dual inhibition of Ang-2 and VEGF-A with faricimab, enhancing treatment durability through agents like brolucizumab or single-administration gene therapies such as RGX-314, and adopting multi-modal management that integrates intravitreal therapies with systemic treatments (e.g., SGLT2 inhibitors) and adjunctive laser modalities. As our understanding of the complex pathophysiology of DME deepens, these innovative and combined strategies will be crucial for optimizing visual outcomes while reducing treatment burden.
